# The impact of “missed nursing care” or “care not done” on adults in health care: A rapid review for the Consensus Development Project

**DOI:** 10.1002/nop2.942

**Published:** 2021-06-16

**Authors:** Eileen Willis, Catherine Brady

**Affiliations:** ^1^ College of Nursing and Health Sciences Flinders University Adelaide Australia; ^2^ Corporate Services Flinders University Adelaide Australia

**Keywords:** adult, adverse events, failure to maintain, missed nursing care, mortality, rapid review

## Abstract

**Aim:**

To identify outcomes of missed nursing care for adult patients.

**Design:**

A five‐stage rapid review process was conducted as follows: refining the question, retrieving relevant studies, determining the studies to be included, organizing the data and synthesizing the results.

**Methods:**

Papers published between 2010–2020 that focused on the UK, Europe, the USA and Oceania were searched for keywords in the title and abstract in major databases. The articles that identified the impact of missed nursing care on adults in health care were selected.

**Results:**

Seventeen articles met the criteria. Major impacts of missed care in adult settings were increases in mortality, adverse events and failure to maintain. These same studies also identified a range of causative factors linked to ward environment, inadequate staffing levels and skills mix although are inconclusive. Solutions include continuing education, ward and work re‐design, and appropriate skill level.

## 1 INTRODUCTION

There is considerable disquiet among citizens in the United Kingdom over the quality of patient care in the National Health Service (Francis, 2013). The University of Sheffield sought to engage citizens in a series of Consensus development discussions in 2020 with the view of generating strategies for reform. The format of Consensus development round tables draws on informed stakeholders and experts to explore a topic (Breart, 1990). The experts are tasked with providing stakeholders with a brief, evidence‐based overview of the issue. This rapid review represents the data provided to those stakeholders considering “missed care for adult patients in acute care.” A rapid review is a simplified systematic review that brings the evidence together in a timely manner but lacks the detailed rigour of systematic reviews. It takes a limited overview of the topic and may reduce the geographical spread and time periods covered and not employ the team‐based procedures of systematic reviews. Its aim was to provide a quick and efficient overview on topics requiring immediate responses (Dobbins, 2017; Haby et al., 2016).

This rapid review explored the question; what is the impact of “missed nursing care” for adult patients? Four outcomes were identified in the research literature, with three addressed here: patient mortality (Schubert et al., 2012), adverse events (Brooks‐Carthon et al., 2016; Liu et al., 2016; Patty et al., 2020) and failure to maintain (Bail & Grealish, 2016). The fourth factor, patient satisfaction is not addressed here. Factors contributing to missed care are also discussed, but only as reported in these papers. A range of current solutions are provided drawing on literature beyond the review.

## 2 BACKGROUND

Missed care is defined by Kalisch (Kalisch et al., 2009, p. 1,510) as any “aspect of required patient care that is omitted (either in part or in whole) or delayed,” while Schubert et al. (Schubert et al., 2012, p. 230) defined “implicit rationing of nursing care” as the “failure to deliver one or more types of needed nursing services.” In reference to patient quality and safety theory, missed or rationed care is seen as an error of omission; something is missed, rather than an error of commission, something is incorrectly given (Kalisch, Landstrom, & Hinshaw, 2009, p. 3). The majority of studies examining missed care are those that ask nurses to estimate the number of times they missed a care task within the last shift or week. The results of these studies show considerable agreement in the list of nursing care tasks left undone (Kalisch, Landstrom, & Hinshaw, 2009). It is assumed that these omitted tasks are detrimental to the patient's health.

Most studies on missed care have been done in the acute hospital sector, in surgical and medical wards and intensive care units (ICU). Nurses are less likely to miss those tasks ordered by doctors such as medication and treatment; hence, it is the *caring tasks* that tend to be left undone more often than treatment or technical tasks. Given these are basic nursing care tasks the question arises as to why this is problematic? One example suffices; poor mouth or oral care is important for preventing teeth loss, gingivitis, and periodontitis for patients who have long‐term care such as those in Care Homes. Failure to maintain adequate mouth care can also be a factor implicated in hospital‐acquired aspirational pneumonia or chest infections (Bail & Grealish, 2016).

## 3 METHOD AND DESIGN

**FIGURE 1:**
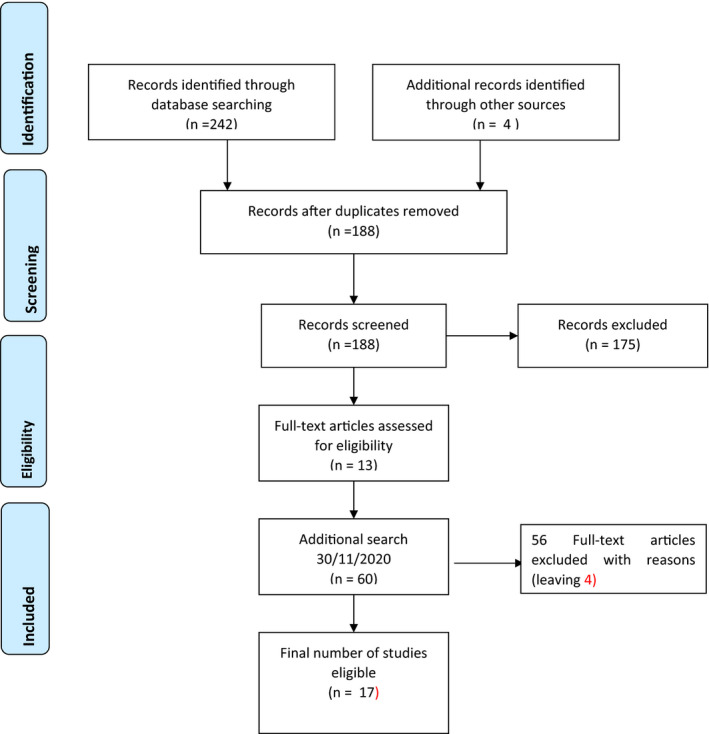
PRISMA record of results (Moher et al., 2009)

A search of keywords was performed using the following keywords (Nurs* AND (“failure to maintain” OR omitted OR “task undone”) AND (“patient safety” OR “patient outcome*”) AND (adult OR adults)) in the search fields of Title & Abstract in the database CINAHL (EBSCOhost) and then translated across to Medline (OVID), Emcare (OVID) and Scopus. The search was filtered by the publication years 01/01/2010 −24/01/2020 and limited to English language. Following the comprehensive search, 242 citations were collated, uploaded into Endnote (Reference management program version X9.2) and duplicates were then removed leaving 184 citations to be appraised. (See PRISMA Figure 1). The search was conducted on 24 January 2020.

An additional four papers were added that were not captured in the search but relevant (Ausserhofer et al., 2013; Ball et al., 2018; Brooks‐Carthon et al., 2015; Lucero et al., 2010). These papers were identified from the reference list of retrieved papers. The paper was drafted by the first author and edited by the second author. The exclusion and inclusion criteria are presented in Table 1. It should be noted that while papers from South Africa, Central and South America, the Middle East and the Eastern Mediterranean Region have been excluded, studies on missed care have been conducted in these countries including Israel, Cyprus and Greece. Cyprus is one of the major leaders in this research domain (Papastavrou et al., 2016). The exclusion was linked to the original brief to focus on the United Kingdom, and the authors own geographical area of interest which includes South East Asia and China.

**TABLE 1:** Inclusion and exclusion criteria

Inclusion	Exclusion
2010 to 1 December 2020	Prior to 2010
Missed care or rationed care in Care Homes/Residential Aged Care, Acute hospital	Paediatric, midwifery, community nursing
Oceania or South East Asia or Europe or UK or North America	South Africa, Central and South America, Middle Eastern Region, Eastern Mediterranean Region
English language	Language other than English
Addresses patient outcomes in article title, abstract	Limits outcomes to impact on nurse, midwife or patient perspective
Provided statistical evidence of mortality, morbidity, adverse event or failure to maintain measured at the same time as missed care	Made claims to possible adverse events or mortality or failure to maintain, but evidence not reported statistically

All articles were read, with 13 papers meeting the criteria and the remaining excluded. An additional search was completed in late 2020 to update the paper for publication. This was done given the proliferation of research in this area over the last four years. Sixty papers were identified with four meeting the criteria, with the first author performing the cull because of her expertise in the area. Of the total 17 studies accepted for this review, nine were cross‐sectional designs, and three were retrospective studies of databases (Table 2) and five were narrative or systematic literature reviews (Table 3). The majority of studies were surveys with nurses self‐reporting care missed on a previous shift.

**TABLE 2:** Studies that associate missed care with specific patient outcomes

No	Study	Mortality	Adverse event	Readmission	Failure to maintain
1	Ausserhofer et al. (2013)		Medication errors Pressure ulcers Falls UTIs HAI NV‐HAP		
2	Bail & Grealish, 2016			Readmissions	UTI Delirium Falls HA Pneumonia
3	Ball et al. 2018	Missed care mediates an association with patient mortality.			
4	Brooks‐Carthon et al. 2016	Association with mortality not established		Readmission 18% higher than other cohorts	
5	Carthon et al 2015			Missed care accounts for 2 to 10% readmissions patients with heart failure	
6	Liu et al. 2016		HAI Surgical site infections Central line blood stream infections UTI VP		
7	Liu et al. 2018		Transfusion site swelling/bleeding Unplanned extubation Phlebitis UTI Transfusion reaction Nosocomial Pneumonia Pressure ulcer Falling injury medication error (infrequent)		
8	Lusero et al 2010		Medication errors HAI Fall with injury infrequently		
9	Nelson et al 2015		UTI		
10	Recio‐Saucedo et al. 2018	Three studies report on correlation between missed care and mortality.		Four studies report on correlation between missed care, readmission and mortality	
11	Schubert et al. 2012	Correlation between patient mortality and missed care and nurse rationing			
12	Tesoro et al., 2018		NV‐HAP correlated with missed care		
13	Zhu et al		Medication error Pressure ulcers Falls Surgical site infection UTI		
14	Patty et al. 2020		Statistically association between bed head elevation and the development of NV‐HAP‐ by 30° to 45° at least twice daily		
15	Ogletree et al. 2020	Death Suicide	Avoidable hospitalization Cardiovascular Events Depression, Falls HAI, UTIs Medication errors Pressure ulcers		Cognitive decline Delirium Functional decline Weight loss
16	Mandal et al. 2020	30 day mortality Postoperative mortality	Medication errors Infections Pressure ulcers Adverse dialysis Falls	Readmission	
17	Labrague et al 2020		HAI, Falls Medication errors		
18	Kalánková et al. 2020		Falls with severe injuries HAIs, UTIs, Medication errors Pressure ulcers.		
Total	18	6	11	5	2

**TABLE 3:** Literature review studies that associate missed care with specific patient outcomes

No	Authors, country and study design	Number of P=patient *N*=nurses H=hospital NH=nursing home	No of studies reporting Mortality	No of studies reporting Adverse Event	No of studies reporting Readmission	No of studies reporting Failure to maintain
1	Bail & Grealish, 2016 Australia Literature review 8 studies	Not reported			Readmissions =8	UTI=8 Delirium=8 Falls=8 HA Pneumonia=8
2	Kalánková et al., 2020 Europe Scoping Literature Review 44 studies	*p* = 1,682 *N* = 119,378	Suicide =1 Postoperative mortality =1	Falls with severe injuries =12 UTIs =1 Medication errors=12 Pressure ulcers =11 Nosocomial infection=13		Deterioration in functional capacity =1
3	Mandal et al. 2020 India Systematic Literature Review 57	*p* = 596,767 *N* = 201,233	30 day mortality=1 Postoperative mortality=1	Nurse reported and hospital reported data records per author Medication errors=2 Nosocomial Infections=1 UTI=1 Pressure ulcers=2 Adverse dialysis=1 Falls =2	Readmission=1	
4	Ogletree et al., 2020 USA Narrative review =327		All‐cause mortality =56 studies Death suicide =7 studies	Avoidable hospitalization=12 Cardiovascular Events=7 Depression=28 Falls=54 Infections=46 HAI, UTIs =14 Medication errors =25 Pressure ulcers =19		Cognitive decline =9 Delirium =9 Functional decline Weight loss =19
5	Recio‐Saucedo et al., 2018 UK Systematic Literature Review	R = 14 *p* = 1,334,666 *N* = 97,000 H = 14,65	Mortality=3	Medication errors=4 Nosocomial infection=4 UTI=1 Pressure ulcers Falls =4	1 report on correlation between missed care, readmission	

## 4 ETHICS

Ethics approval for this study was not required as no individuals including patients were included. Funding was received from the University of Sheffield.

## 5 RESULTS

These are (i) mortality rates; (ii) adverse events; (iii) and failure to maintain.

### 5.1 Acute hospital sector

#### 5.1.1 Mortality rates

Three papers demonstrated a statistical link between mortality outcomes and missed care (Ball et al., 2018; Schubert et al., 2012; Tesoro et al., 2018) with two other studies finding no statistical links (Brooks‐Carthon et al., 2016; Lucero et al., 2010). Three literature reviews reported on mortality rates, citing some of the papers listed above, but the authors came to varying conclusions; Recio‐Saucedio et al. reported on the differences across studies, while Mandal and Ogletree concluded the evidence supported a link (Mandal et al., 2020; Ogletree et al., 2020; Recio‐Saucedo et al., 2018). For example, Schubert et al. examined the association between explicit rationing and hospital mortality in medical, surgical and gynaecological wards demonstrating that patients treated at hospitals with the highest rates of missed care have a 51% increase in mortality (Schubert et al., 2012). These results were substantiated in a study by Ball et al. (2018) across 300 acute hospitals in nine European countries. As missed care increased, so too did case mix adjusted mortality rates within 30 days of admission (Ball et al., 2018). The less conclusive findings on patient mortality reported by Recio‐Saucedo et al., identified four studies that explored the relationship between mortality and missed care (Ambrosi et al., 2016; Brooks‐Carthon et al., 2016; Lucero et al., 2010; Schubert et al., 2012). The Ambrosi et al., paper is not included here as the missed care was recorded by relatives. Recio‐Saucedo et al., conclude that only the study by Schubert et al., returned reliable results noting that once adjustments were made for patients, ward or hospital environmental factors the results were not statistically significant.

#### 5.1.2 Adverse events

Seven studies reported on adverse events, with five literature reviews repeating some of these results (Tables 2 and 3). Adverse events such as medication errors, urinary tract infections, patient falls, pressure ulcers, critical incidents, and poor quality of care arising from missed care were reported across a number of studies (Lucero et al., 2010; Recio‐Saucedo et al., 2018), while readmissions were also reported (Brooks‐Carthon et al., 2015). A small number of studies note specific adverse events such as increases in bloodstream infections and pneumonia (Ausserhofer et al., 2013; Tesoro et al., 2018) or statistically significant outcomes for medication errors, infections, pressure ulcers, dialysis events, falls and readmissions (Mandal et al., 2020).

Tesoro et al., (2018) studied the number of patients in the state of New York in 2014 who were not mechanically ventilated and contracted non‐ventilated hospital‐acquired pneumonia (NV‐HAP). Of the 837 patients who contracted NV‐HAP, the following nursing care tasks were missed; 50% of patients did not have their bed elevated, one third were not assisted to walk around each day, fewer than 16% had had any deep breathing exercises to keep their chest clear, fewer than 20% had incentive spirometry and less than 49.5% had any mouth care. Forty‐four per cent of these 837 patients were discharged to a care home either permanently or for rehabilitation. The mean age of the patients was 64 years, and the majority were Black Americans (34.1%). Their Average Length of Stay in hospital was 24 days and 25% were readmitted within 30 days. In a similar US study situated within a community hospital in California Patty et al., (2020) reported that elevated bed heads reduced NV‐HAP by 26%, but there was no association with mouth care, ambulation, deep breading exercises, use of spirometry or mouth care (Patty et al., 2020). The difficulty in establishing direct causation between missed care and adverse events is spelt out by Kalánková et al., who distinguish between the risk of adverse events and direct evidence/causation. In their systematic literature review, they cite 25 papers that make claim to direct adverse outcomes; however, papers go back to 2001, and a number of the studies rely on nurse perceptions, rather than evidence of direct causation (Kalánková et al., 2020).

The relationship between missed care and adverse events has also been researched in the Asian region (Zhu et al., 2019). Zhu et al., sought to establish a connection between staffing levels and rationed nursing care, arguing that lower staffing levels would result in higher numbers of care tasks missed, and higher numbers of adverse events. Using two survey methods, one based on the Basil Extent of Rationing of Nursing Care (BERNCA) (Schubert et al., 2007), but adapted for China, and a patient satisfaction survey, they explored the relationship between nurse staffing and missed care and found that 68% of nurses reported at least two to four tasks were missed. This study is particularly pertinent as these nurses reported that patients suffered adverse events. While the authors did not link particular missed care to an adverse event there is the suggestion of a linkage or pathway. While rates of adverse events were low, as expected, the authors suggested a mediating relationship between staffing levels and adverse events. In an earlier study in China Liu et al., examined the relationship between missed care, adverse events and the overall hospital environment. They once again noted that there was a pathway between the working environment for nurses within the hospital and adverse events, with missed care being the intervening factor (Liu et al., 2018).

#### 5.1.3 Patient characteristics, missed care and adverse events

There is some suggestion in the research literature that patient characteristics make a difference to the number of care tasks that are missed and subsequent adverse events. This is tackled in two ways. Studies may adjust the findings based on the case mix, or characteristic of the patient, before making a definitive statement about the quality of the care provided given some patients are more vulnerable than others (Ball et al., 2018). The second approach is to identify these characteristics and see if these patients receive inferior care. Race is one category examined in the USA (Brooks‐Carthon et al., 2016). Brooks‐Carthon et al. (2016) examined the rate of readmissions within 30 days for Black American patients following an acute myocardial infarction (AMI). The question posed was whether or not readmission rates were due to epidemiological characteristics of the population, or hospital factors such as missed nursing care, staff‐patient ratios or the hospital work environment. Six per cent of patients were Black‐American, but their readmission rate was 23.5% over 18.8% for Caucasian patients, despite the Black‐American patients being younger. They had higher numbers of comorbidities and were of a lower socioeconomic status (SES). Black‐American patient numbers were higher in hospitals that did poorly on nurse‐reported surveys on the work environment, although these hospitals were better staffed. While the authors admit that one of the underlying reasons for readmission may be the patient's lower SES which may impact on their access to specialist post‐hospital care, including access to medications; the relationship between documentation and patient communication is seen as a key explanatory factor along with timely medication which had the strongest association with readmission rates per hospital. A more recent study conducted in California with patients with several comorbidities indicated that they were less likely to experience an adverse event. In this case the authors argued that nurses were less likely to miss care where they knew the patient was vulnerable (Patty et al., 2020).

#### 5.1.4 Failure to maintain leads to adverse events

The concept of “failure to maintain” coined by Bail (Bail & Grealish, 2016) traces the relationship between a sequence of missed care tasks, or mild neglect, and the onset of an adverse event for the frail elderly through a process known as cascade iatrogenesis. Failure to maintain is defined as “insufficient delivery of essential nursing care for an older person in hospital resulting in a complication, four of which are useful indicators for the quality of hospital performance particularly for patients with dementia” (Bail & Grealish, 2016, p. 153). In coining the term “failure to maintain,” Bail is deliberately linking it to failure to rescue (death following a hospital‐acquired adverse event). Ogletree et al., identified cognitive and functional decline, delirium and weight loss resulting from missed care (Ogletree et al., 2020) and Kalánková et al. (2020) in functional capacity.

### 5.2 Care Homes

#### 5.2.1 Adverse event/readmissions

Less research has been done within the care homes for older people. As Ogletree et al., noted in their systematic review, studies are inconsistent in reporting adverse events with some noting potential, but few providing direct evidence (Ogletree et al., 2020). Despite this caveat, Ogletree et al., define 14 outcomes as adverse events, although the study source is not identified. However, they do identify six papers that report a correlation between missed care and adverse events in aged care. Of the six papers they identified, two met the criteria for this rapid review. The other four papers included community‐based patients making it difficult to distinguish residents in aged care homes (Nelson & Flynn, 2015; Recio‐Saucedo et al., 2018).

Nelson and Flynn (2015) explored the relationship between missed care and adverse events in nursing homes in the USA, drawing on nationally collected data obtained through the Online Survey Certification and Reporting (OSCAR) database and the Minimum Data Set (MDS) for Medicare and Medicaid funded homes in the USA. Drawing on data from 63 nursing homes and 340 RNs, they found that failure to administer medications on time and to provide adequate surveillance had the strongest association with the incidence of Urinary Tract Infections (Nelson & Flynn, 2015).

## 6 DISCUSSION: CAUSES OF MISSED CARE

There is difficulty in establishing evidence to support claims that missed care leads directly to increased mortality, adverse events or failure to maintain given most studies are surveys that report on nurse's subjective observations. It is also difficult to establish the direct pathway between forgetting to assist a patient to ambulate and aspirational pneumonia, or any other adverse event. Some studies reported here have also asked the respondents to rate the nursing working culture and to report on the nurse–patient ratios at the time of doing the missed care survey (Liu et al., 2016, 2018; Lucero et al., 2010). The work culture is invariably measured using the nurse work environment index, which asks nurses their views on staffing levels and resources, collegial relationships with doctors, their nurse manager's ability to lead and support the team and participation in hospital affairs. The nurse–patient ratio is measured by the number of nurses rostered on the shift against the number of patients (Schubert et al., 2012). For example, Schubert's study on the association between explicit rationing of care and mortality rates used both approaches showing a correlation between the nursing environment, staffing levels, missed care and mortality rates, but this does not establish a direct causation, just a correlation. What is known is that good working environments lead to highly satisfied nurses who are less likely to miss care, resulting in better patient outcomes (Smith et al., 2018).

Bail and Grealish (2016) cites increased patient throughput, acuity, comorbidity and disability along with the relentless healthcare systems and hospital focus on efficient length of stay, as factors that result in failure to maintain. She refers to this as the “disappearance of recovery time” (Bail & Grealish, 2016, p. 151). Coupled with this is an argument that there are too few experienced nurses working directly in clinical positions, that inexperienced and younger staff are rostered on after‐hours and weekends constituting 75% of the hours worked over the week and that nursing work is highly fractured with high levels of interruptions over the course of the shift requiring very clear organizational and time management skills (Bail & Grealish, 2016).

Few studies examine nurse characteristics, or report on any specific nurse qualities, other than to confirm that the population being surveyed mirrors that of the total nurse population (Schubert et al., 2012). Some papers note the number of nurses who have a university qualification (Ball et al., 2018; Zhu et al., 2019), but there is no consistent findings on the relationship between nurse education or other characteristics and patient outcomes. A number of publications examine managerial leadership style but are not included in this review (Chapman et al., 2017; Srulovici & Drach‐Zahavy, 2017). Similarly, there are variations in nurse reporting of missed care based on experience and ethnicity, but not necessarily linked to specific patient outcomes (Blackman et al., 2015). In summary, the research on missed care remains inconclusive in terms of mortality, adverse events or failure to maintain but does suggest a correlation between patient outcomes and nursing omissions. The variety of papers identified in this rapid review also point to missed care being a global phenomena.

## 7 CONCLUSION: POLICY IMPLICATIONS

The majority of studies identify resource and staffing shortfalls as the primary cause of missed care (Schubert et al., 2012). Research demonstrating the relationship between patient morbidity and mortality as a result of nurse staffing levels is now over twenty years in the making (Aiken et al., 2002). Despite this, the evidence on what nurse staffing levels should be remains elusive. As a consequence, policy has moved in two direction, particularly in the Anglo countries of the UK, USA, New Zealand, Australia and Canada. The first has been attempts to see missed care as a managerial or motivational issue and to address the problem through ward‐based re‐designs (for example, Lean production systems (Attwood‐Charles & Babb, 2017), projects aimed at enhancing nurse compassion (O’Driscoll et al., 2017) or to go back to the fundamental of nursing care (Kitson et al., 2014)).

### 7.1 Limitations and implications

There are two major methodological limitations in this rapid review. Rapid reviews are a limited approach to compiling the available evidence on a topic. They are performed quickly, may limit the parameters of the search and protocols employed and as a consequence risk missing important studies. For example, this rapid review omits a number of studies from countries that have produced significant work, for example Cyprus (Papastavrou et al., 2014) and focuses on Asia/Oceania. This reflects the first author's interest, and a desire to demonstrate the spread of the phenomena beyond Europe and the USA and to illustrate that no matter what healthcare systems are in place, nursing care is missed. Secondly, the copious research conducted on missed care, particularly over the last 4 years has raised a number of problematic issues (Vincelette et al., 2019). The first is the way the research data is gathered. The majority of studies ask for nurse's subjective observations of missed care, either during the last shift, or the last week (Kalisch et al., 2009), and all assume that the tasks are the sole responsibility of nurses. Little research has tested rates of missed care through ethnographic observations (Lake et al., 2016), or through recognized forms of documentation (Tesoro et al., 2018). Alternative research approaches may provide a more reliable evidence base than nurses subjective records. Further, hospitals are staffed with more than nurses, yet little research has explored the impact other health professionals, such as the medical or allied health staff, may or may not have on rationed care.

## CONFLICT OF INTERESTS

There are no competing interests.

## AUTHOR CONTRIBUTIONS

Eileen Willis: Conception of the study and manuscript writing, with the original and updated searches completed by Catherine Brady.

## Data Availability

The process of data retrieval is outlined in the method section and the Endnote library is available from the authors at Eileen.willis@flinders.edu.au.
